# Association of Fordyce's Granules with hyperlipidemia: A clinical indicator of lipid profile alterations

**DOI:** 10.6026/973206300212767

**Published:** 2025-08-31

**Authors:** Ashutosh Pandey, Sidhant Kumar, Asmita Kumar, Ajeet Kumar, Anjana Bharti, Sagar Mani, Siddharth Saurabh, Manoj Mittal

**Affiliations:** 1 Department of Dentistry, Anugrah Narayan Magadh Medical College and Hospital, Gaya, Bihar, India; 2 Department of Dentistry, Nalanda Medical College and Hospital, Patna, Bihar, India; 3 Department of Dentistry, Netaji Subhash Medical College and Hospital, Bihta, Bihar, India; 4 Department of Conservative Dentistry and Endodontics, Mithila Minority Dental College and Hospital, Darbhanga, Bihar, India; 5 Private Practitioner, Buddha Dental Hospital, Patna, Bihar, India; 6 Department of Oral Medicine and Radiology, Private Practitioner, Your dentist, Kashi Heights, Ujala Complex Jhumritelaiya, Jharkhand, India; 7 Department of Oral Medicine and Radiology, Bhabha College of Dental Sciences (Bhabha University), Bhopal, Madhya Pradesh, India; 8 Department of Periodontology (Ex Dean), Bhabha College of Dental Sciences (Bhabha University), Bhopal, Madhya Pradesh, India

**Keywords:** Fordyce's granules, oral mucosa, hyperlipidemia, lipid profile, cardiovascular risk

## Abstract

Fordyce's granules (FG) are ectopic sebaceous glands that are usually localized in the oral mucosa. Hence, we compared 150 patients with FGs and 100 patients without, the results being based on the lipid profile after fasting. Findings showed that the most common sites were the buccal mucosa and higher FGs were found in individuals who were male in the age of 20-40. When the FG presentation was dense (>100 granules), the results depicted in the total cholesterol and LDL were highly altered. FGs can be considered as one of the possible early clinical predictors of hyperlipidemia and cardiovascular risk.

## Background:

Fordyce granules (FGs) or Fordyce spots or ectopic sebaceous glands are harmless, heterotopic sebaceous glands that appear in the mouth, especially in the buccal mucosa and vermilion border of the lips. They present as asymptomatic small papules of yellowish or whitish colour measuring about 1 to 3mm in diameter, without cutaneous follicular relation [1]. They are regarded as a normal anatomical variation, but in recent literature, their presence is studied with regard to its clinical implications because of their possible association with systemic metabolic changes [2]. Atherosclerosis and coronary artery disease are largely susceptible to a major risk factor, which is hyperlipidemia, which is a condition characterized by the elevation of lipids in the blood. Early screening and treatment of the abnormalities of lipid is an important step in preventing cardiovascular occurrences [3]. Lipid metabolism disturbances have been linked to a number of dermatological and mucosal manifestation indicators. Fordyce granules in particular have been cited as possibly being a clinical specimen, particularly in large quantities [4]. It is considered that the pathophysiological pathway between FGs and lipid metabolism resides in the activity of the sebaceous glands affected by the level of serum lipids and hormonal components [5]. Research studies also indicate that higher sebaceous activity and the overgrowth of sebaceous glands may be evidence of subterranean dyslipidemia [6]. As Fordyce granules can easily be diagnosed upon the normal inspection of the mouth, they can be used as one of the easily available clinical signs of abnormalities of lipids, therefore, this finding concurs with the hypothesis that even hyperplasia of sebaceous glands in ectopic locations can be indicative of the overall lipid metabolism imbalances [7]. Sebaceous glands are recognized to be lipid-secreting organs and the metabolic activity of these glands is immensely dependent on the level of circulating lipids and androgenic hormones. Lipids produced by sebocytes are triglycerides, wax esters and squalene and these resemble systemic lipid composition in some ways [8]. Thus, ectopic sebaceous glands of the oral mucosa, including Fordyce granules, may appear more or even numerous in the state of lipid dysregulation. With the rising incidence of cardiovascular diseases attributed to dyslipidemia, the prospect of a non-invasive, low-cost clinical screening tool with the ability to produce an early warning with the help of the Fordyce granules would only improve the effectiveness of earlier detection and interventional measures [9]. Research has revealed that FGs are more common among men and usually manifest themselves in their second to fourth decades of life, which happens to be the time when people are at risk of getting lipid abnormalities. This overlapping age- and sex-associated distribution indicates that the granules of Fordyce might become part and parcel of the expanded clinical investigations of cardiovascular risk profiling, particularly in large populations and/or at highly predictable sites within the mouth cavity [7, 8- 9]. Therefore, it is of interest to evaluate the accepted notion that the occurrence and load of oral Fordyce granules could be related to unusual serum lipid levels to present a non-invasive indicator of early hyperlipidemia and cardiovascular risks.

## Materials and Methods:

A total of 250 individuals, aged 20 to 70 years, were enrolled. Among them, 150 subjects clinically exhibited Fordyce's granules (study group), while 100 subjects with no visible Fordyce's granules served as the control group. Individuals with systemic disorders, ongoing lipid-lowering therapy, or habits such as tobacco and alcohol use were excluded to avoid confounding effects. The diagnosis of Fordyce's granules was made through clinical examination under adequate illumination, identifying small, painless, yellowish or whitish papules typically located on the buccal mucosa and lips. The granules were recorded and categorized based on their location (L1-L6: lips, buccal mucosa, retromolar area, etc.) and density (D1: 1-20, D2: 21-50, D3: 51-100, D4: >100 granules). All participants were instructed to undergo an overnight fast for 12 hours before venous blood was drawn. Serum lipid profile was assessed, including total cholesterol (TC), triglycerides (TG), low-density lipoprotein (LDL) and high-density lipoprotein (HDL) using standard enzymatic colorimetric methods in a certified biochemistry laboratory. Statistical analysis was performed using SPSS software. Comparative analysis between groups was done using an independent sample t-test for continuous variables and a Chi-square test for categorical data. Analysis of variance (ANOVA) was used to evaluate the difference in lipid levels across FG density groups. Cramer's V test was applied to assess the strength of association between FG presence and lipid levels. A p-value of <0.05 was considered statistically significant.

## Results:

A total of 250 participants were included in the study, comprising 150 individuals with Fordyce's granules (FG group) and 100 individuals without Fordyce's granules (control group). The participants were evaluated based on demographic characteristics, FG location and density and their serum lipid profiles. Among the FG group, 65.3% were males, while in the control group, 58% were males. The most common age group with Fordyce's granules was between 21-40 years, accounting for 56% of cases (Table 1 - see PDF). There was a statistically significant difference in the age distribution between the groups (p < 0.05). The buccal mucosa was the most frequently affected site (78.6%), followed by the upper lip (52.6%). In terms of distribution, bilateral presentation was more common than unilateral. Regarding density, 28% of participants had more than 100 granules (D4) and this group showed a tendency toward elevated lipid levels (Table 2 - see PDF). Significant differences were observed in lipid parameters between groups. The FG group showed elevated mean total cholesterol and LDL-C levels when compared to the control group (Table 3 - see PDF). The difference in HDL and triglyceride levels was not statistically significant. As illustrated in Tables 3 (see PDF) and 4 (see PDF), individuals with a higher density of Fordyce's granules (particularly D4) consistently demonstrated elevated total cholesterol and LDL-C levels, suggesting a positive association between FG severity and hyperlipidemia risk.

## Discussion:

Fordyce granules (FGs), which tend to be harmless and asymptomatic, are of interest to clinicians as they may be linked with systemic metabolic diseases like hyperlipidemia. The current study also confirms the statistically sound relationship between the occurrence and abundance of FGs and increased serum lipid levels, especially total cholesterol (TC) and low-density lipoproteins (LDL) and hence proves the hypothesis that the mucosal sebaceous structures could be used as outer markers of lipid imbalances [1]. The findings of the age distribution of FGs in males in the study and higher FGs in the 2040 years category do support the findings of previous studies attributable to androgen impact on the activity of the sebaceous gland [2]. The effect of androgens on the induction of sebocyte growth and lipid production may indicate the high occurrence and the larger size of FGs in the hormonally active times [3]. The most common sites of FG manifestation were still the buccal mucosa and the upper lip and were filled with the previously demonstrated anatomical patterns in terms of the distribution of oral pathology [4]. Notably, we have found that the abundance of Fordyce granules in the population (normal cases with less than 100 papules and patients with levels higher than 100 papules) was markedly increased by the TC and LDL levels. This concurs with the findings of Shetty *et al.* and Chaudhary *et al.* who found that there was a high prevalence of having numerous FG densities in combination with lipid abnormalities [5, 6]. The biological correlates of the relationship are that the sebaceous gland is involved in the metabolism of lipids. The sebocytes produce sebum, which is highly enriched by cholesterol and its derivatives and their production depends immediately on systemic lipid levels [10]. Also, sebaceous glands themselves, as well as the heterotopic sebaceous glands, react to the systemic dyslipidemia by elevating the production of lipids and proliferation of the cells. The regulation of these responses is guided by peroxisome proliferators-activated receptor (PPARs) and sterol regulatory elements-binding proteins (SREBPs) that dictate the systemic and the sebaceous lipid processing [11]. Hence, the raised levels of lipids can cause hypertrophy or growth of the oral sebaceous glands, causing them to become clinically visible in the form of Fordyce granules [12]. The diagnostic verification of FGs as a risk marker of hyperlipidemia among people is of great importance to the population. Dental offices typically administer oral tests and the increased manifestation of FGs may be one of the early non-invasive symptoms that lead to the examination of the lipid profile, even in patients who do not present with the obvious cardiovascular distress [13]. This is concurrent with the wider revolution in predictive medicine, in which visible phenomena (xanthelasma or acanthosis nigricans) can be used as early warning signs of essentially systemic metabolic disease [14]. In our study, HDL and triglyceride levels were not significantly different between groups, but trends indicate that further study of larger sample sizes and experimental controls might yield small effects. The non-standard or random results obtained between FGs and serum triglycerides have also been reported in other studies, presumably because of the distinct metabolism of FGs with LDL and cholesterol [15]. It can also be mentioned that the oral mucosa has been traditionally regarded as the window to systemic health. Mucosal pallor, petechiae or candidiasis are other common clinical signs that are related to anemia, bleeding disease or immunodeficiency respectively [16]. Applying this idea further, the granules of Fordyce could become an oral indicator of lipid dysfunction and inclusion into routine oral-systemic health screening would help to raise the early detection rates [17]. These positive results notwithstanding, there are limitations to this study. It is cross-sectional and, therefore, it leaves no chance to determine the causality between FG density and lipid abnormalities. Also, factors like genetics, diet, exercise and even hormonal status did not receive any control and could have affected the lipid profile [18]. It needs to be studied in longitudinal cohort studies whether persons with high FG density have an increased risk of developing cardiac outcomes with the progression of time.

## Conclusion:

The granules that are associated with the elevated levels of serum lipids in large proportions are the granules of Fordyce. The positive association of periodic oral tests with the insidious nature of subsequent hyperlipidemia can be used as a non-invasive clinical parameter. Small identification would help in the assessment of cardiovascular risk and prevention in healthcare.
